# Usutu Virus Persistence and West Nile Virus Inactivity in the Emilia-Romagna Region (Italy) in 2011

**DOI:** 10.1371/journal.pone.0063978

**Published:** 2013-05-07

**Authors:** Mattia Calzolari, Paolo Bonilauri, Romeo Bellini, Alessandro Albieri, Francesco Defilippo, Marco Tamba, Massimo Tassinari, Antonio Gelati, Paolo Cordioli, Paola Angelini, Michele Dottori

**Affiliations:** 1 Istituto Zooprofilattico Sperimentale della Lombardia e dell’Emilia Romagna “B. Ubertini” (IZSLER), Brescia, Italy; 2 Centro Agricoltura Ambiente “G. Nicoli” (CAA), Crevalcore, Italy; 3 Servizio Veterinario Azienda USL Ferrara, Ferrara, Italy; 4 DG Sanità e Politiche Sociali, Regione Emilia-Romagna, Bologna, Italy; Blood Systems Research Institute, United States of America

## Abstract

**Background:**

The circulation of West Nile virus and Usutu virus was detected in the Emilia-Romagna region in 2008 and 2009. To evaluate the extent of circulation of both viruses, environmental surveillance, based on bird and mosquito testing, was conducted in 2008 and gradually improved over the years.

**Methods:**

In February–March 2009–2011, 5,993 hibernating mosquitoes were manually sampled, out of which 80.1% were *Culex pipiens*; none tested positive for the viruses. From 2008 to 2011, 946,213 mosquitoes, sampled between May and October, were tested; 86.5% were *Cx. pipiens*. West Nile virus was detected in 32 *Cx. pipiens* pools, and Usutu virus was detected in 229 mosquito pools (217 *Cx. pipiens*, 10 *Aedes albopictus*, one *Anopheles maculipennis s.l.*, and one *Aedes caspius*). From 2009 to 2011, of 4,546 birds collected, 42 tested positive for West Nile virus and 48 for Usutu virus. West Nile virus and Usutu virus showed different patterns of activity during the 2008–2011 surveillance period. West Nile virus was detected in 2008, 2009, and 2010, but not in 2011. Usutu virus, however, was continuously active throughout 2009, 2010, and 2011.

**Conclusions:**

The data strongly suggest that both viruses overwinter in the surveyed area rather than being continually reintroduced every season. The lack of hibernating mosquitoes testing positive for the viruses and the presence of positive birds sampled early in the season support the hypothesis that the viruses overwinter in birds rather than in mosquitoes. Herd immunity in key bird species could explain the decline of West Nile virus observed in 2011, while the persistence of Usutu virus may be explained by not yet identified reservoirs. Reported results are comparable with a peri-Mediterranean circulation of the West Nile virus lineage 1 related strain, which became undetectable in the environment after two to three years of obvious circulation.

## Introduction

West Nile virus (WNV) was first reported in the Emilia-Romagna region in 2008 [1], and evidence of circulation was obtained until 2010 [2]. The high identity from partial nucleotide sequencing of positive mosquitoes, humans, and wild birds suggests that the virus overwintered rather than being re-introduced several times [2], [3], [4].

Usutu virus (USUV) has been detected in the same area since 2009 [3], [5], and biomolecular data demonstrate that the virus has overwintered in Emilia-Romagna [2]. This flavivirus was first reported in Europe in Vienna in 2001 [6]; from there, the virus was reported in neighboring countries and has become endemic [7]. Both viruses belong to the Japanese encephalitis antigenic complex [8] and are phylogenetically closely related [9], but while WNV is a known pathogen for humans, the pathogenicity of USUV to humans is not fully understood [10], [11].

Similar to other arboviruses (vector-borne viruses), WNV and USUV exploit complex biotic and trophic interactions between vectors and reservoirs, to persist in a particular area. These interactions are influenced by numerous ecological and environmental factors that can confer unique characteristics on the virus cycle in a particular ecosystem, including differential ways of transmission and permanence. Due to this variability, characterizing the epidemiological picture in a specific environment, identifying all transmission ways and discovering the predominant ones, is difficult. This is especially true for WNV, which may exploit many arthropod vectors and reservoir species [12]. Certainly, wild birds are the most significant reservoir species for WNV, with different viremia duration and intensity according to the species [13], and a different role in virus transmission according to the species abundance. The USUV cycle, however, is less well known but seems to be similar to the WNV cycle.

Overwintering is a critical point for virus permanence in temperate regions. Several mechanisms have been hypothesized to explain overwintering of arboviruses. When the vector survives from one season to the next by arresting development and reducing metabolism (diapause), as in the case of the *Culex pipiens* mosquito, the virus can persist in the adult vector. The virus can also survive the winter in the vector eggs, or more rarely, in the juvenile stages, when they are the diapausing stages [14].

Vertebrates can also contribute to arbovirus overwintering. For example, a delayed viremia can be produced in hibernating animals at their awakening, or in some specimens a recurrent or long-lasting viremia could be produced, or the virus could persist in specific organs of animals, and be reactivated after ingestion by predators or scavengers [14], producing an infection.

Since 2008, an environmental surveillance program targeted toward WNV and USUV has been active in the Emilia-Romagna region [15], [16]. This system has been improved over the years, and this paper reports the 2011 monitoring results. To explain the permanence mechanisms of the two viruses in the region, the data obtained in previous years [2], [3], [5], [16] were also analyzed retrospectively.

## Results

### Surveillance results for 2011

#### Mosquitoes

From February 11 to March 14, 4,109 overwintering mosquitoes were sampled manually from winter hibernacula (of which 3,524, equal to 80.1%, were *Cx. pipiens*) (Table 1). A particular effort was made in the Modena province, where 2,333 mosquitoes were collected at one site on three sampling days (1985 *Cx. pipiens*, 348 *An. maculipennis s.l.*), and 699 *Cx. pipiens* specimens were collected at another site during one day.

**Table 1:** Overwintering mosquitoes collected between 2009 and 2011.

	2009			2010			2011			Total		
Species	*n*	%	pools	*n*	%	pools	*n*	%	pools	*n*	%	pools
*An. maculipennis s.l.*	475	47.9	14	129	14.4	21	585	14.2	40	1189	19.8	75
*Cs. annulata*	4	0.4	2	1	0.1	1		<0.1		5	0.1	3
*Cx. pipiens*	512	51.7	24	764	85.5	52	3524	85.8	97	4799	80.1	172
Total	991		40	894		74	4109		137	5993		250

At three sites, by using carbon dioxide attraction traps weekly from March 10 to May 20, 258 mosquitoes were collected, of which 252 (97.7%) belonged to the species *Cx. pipiens* (Table 2).

**Table 2:** Early season-sampled mosquitoes collected in 2009 and 2011.

	2009			2011			Total			
Species	*n*	%	pools	*n*	%	pools	*n*	%	pools	
*Ae. albopictus*	0	0	0	1	0.4	1	1	0.0	1	
*Ae. caspius*	384	14.9	26	3	1.2	1	387	13.6	27	
*Ae. detritus*	157	6.1	5				157	5.5	5	
*An. maculipennis s.l.*	2	0.1	2	2	0.8	2	4	0.1	4	
*Cs. annulata*	2	0.1	2				2	0.1	2	
*Cx. pipiens*	2040	78.9	55	252	97.7	29	2292	80.6	84	
Total	2585		90	258		33	2845			

**Figure 1:**
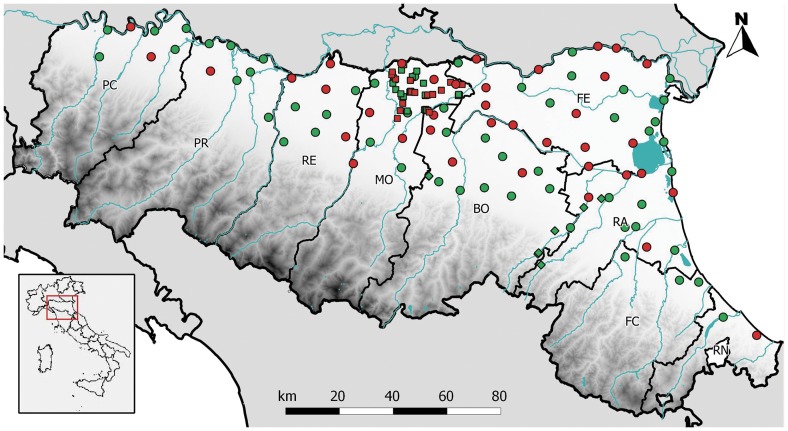
Emilia-Romagna regional map with mosquito sampling stations and locations of USUV-positive pools. Circle, plan trap; square, Modena trap; diamond, extra-plan trap; red, USUV-positive station; green, negative station. Province abbreviations: PC, Piacenza; PR, Parma; RE, Reggio Emilia; MO, Modena; BO, Bologna; FE, Ferrara; RA, Ravenna; FC, Forlı ` Cesena; RN, Rimini.

From May 31 to October 19, a total of 269,686 mosquitoes, grouped in 2,451 pools, were collected with carbon dioxide (CO2) baited traps and analyzed; 227,754 specimens were sampled with plan traps, 41,316 were sampled with Modena traps, and 616 were sampled with extra-plan traps. The sites sampled are shown in Figure 1. The most abundant species was *Cx. pipiens* (86.4% of tested mosquitoes), followed by *Aedes caspius* (10.2%), *Ae. vexans* (2.3%), and *Ae. albopictus* (0.9%) (Table 3). Other species sampled, but at low density (under 0.2%), were *Anopheles maculipennis s.l.*, *Cx. modestus*, *Coquillettidia richiardii*, and *An. plumbeus* (Table 3).

**Table 3:** Mosquitoes sampled from May 31 to October 19, 2011.

	*n*	%	pools	USUV/+
*Ae. albopictus*	2,450	0.9	192	6
*Ae. caspius*	27,387	10.2	466	1
*Ae. vexans*	6,140	2.3	114	
*An. maculipennis*	286	0.1	23	1
*An. plumbeus*	5	0.0	3	
*Cq. richiardii*	143	0.1	4	
*Cx. modestus*	201	0.1	15	
*Cx. pipiens*	233,074	86.4	1,634	74
Total	269,686		2,451	82

Several sites were sampled in 2010 and 2011, and included 80 plan trap stations and 31 Modena trap stations. For both trap groups, the number of *Cx. pipiens* mosquitoes per night was higher in 2010 than in 2011. The average number of mosquitoes/trap/night was 707.6 (1,156.7 s.d.) in 2010 and 340.9 (570.1 s.d.) in 2011 for the plan traps (p<0.01) and 266.2 (171.9 s.d.) in 2010 and 114.0 (67.3 s.d.) in 2011 for the Modena traps (p<0.01).

#### Birds

A total of 1,068 wild birds, collected within the specific wildlife population control program between January and November, were tested. Three corvid species were the most represented species: Eurasian Magpies (*Pica pica*, 558 specimens), Hooded Crows (*Corvus cornix*, 415 specimens), and Eurasian Jays (*Garrulus glandarius*, 68 specimens). Moreover, 26 European Starling (*Sturnus vulgaris*) were actively collected (Table 4).

**Table 4:** Birds tested in active and passive surveillance (A/P) in 2011 with USUV-positive specimens.

Name	Species	Collected specimens (A/P)	USUV/+ specimens (A/P)
Eurasian Magpie	*Pica pica*	582 (558/24)	14 (13/1)
Hooded Crow	*Corvus cornix*	421 (415/6)	0
Eurasian Jay	*Garrulus glandarius*	81 (68/13)	1 (1/0)
Eurasian Collared-Dove	*Streptopelia decaocto*	40 (0/40)	1 (0/1)
Eurasian Blackbird	*Turdus merula*	37 (0/37)	1 (0/1)
Common Starling	*Sturnus vulgaris*	32 (26/6)	2 (0/2)
Yellow-legged Gull	*Larus michahellis*	25 (0/25)	1 (0/1)
House Sparrow	*Passer domesticus*	12 (0/12)	1 (0/1)
Eurasian Green Woodpecker	*Picus viridis*	9 (0/9)	1 (0/1)
European Bee-eater	*Merops apiaster*	3 (0/3)	1 (0/1)
Grey Heron	*Ardea cinerea*	5 (0/5)	1 (0/1)
Greater Spotted Eagle	*Aquila clanga*	1 (0/1)	1 (0/1)
Other birds		238 (1/237)	0
Total		1486 (1068/418)	25 (14/11)

Common Kestrel (*Falco tinnunculus*) 35, Little Owl (*Athene noctua*) 29, Common Pigeon (*Columba livia domestica*) 21, Black-headed Gull (*Larus ridibundus*) 15 (one specimen actively collected), Common Swift (*Apus apus*) 14, Long-eared Owl (*Asio otus*) 14, Common Buzzard (*Buteo buteo*) 10, Mallard (*Anas platyrhynchos*) 9, Common Pheasant (*Phasianus colchicus*) 8, Eurasian Sparrowhawk (*Accipiter nisus*) 6, Barn Swallow (*Hirundo rustica*) 6, Little Bittern (*Ixobrychus minutus*) 6, Eurasian Scops Owl (*Otus scops*) 6, Eurasian Nightjar (*Caprimulgus europaeus*) 5, Great Tit (*Parus major*) 4, Cattle Egret (*Bubulcus ibis*) 3, Eurasian Jackdaw (*Corvus monedula*) 3, Great Crested Grebe (*Podiceps cristatus*) 3, Northern House-Martin (*Delichon urbica*) 2, Great Spotted Woodpecker (*Dendrocopos major*) 2, Little Egret (*Egretta garzetta*) 2, European Robin (*Erithacus rubecula*) 2, Indian Peafowl (*Pavo cristatus*) 2, Great Cormorant (*Phalacrocorax carbo*) 2, Barn Owl (*Tyto alba*) 2, Northern Goshawk (*Accipiter gentilis*) 1, Eurasian Skylark (*Alauda arvensis*) 1, Common Teal (*Anas crecca*) 1, Goose (*Anser anser*) 1, Pallid Swift (*Apus pallidus*) 1, Squacco Heron (*Ardeola ralloides*) 1, Great Bittern (*Botaurus stellaris*) 1, European Goldfinch (*Carduelis cardueli*) 1, Black Tern (*Chlidonias niger*) 1, White Stork (*Ciconia ciconia*) 1, Eurasian Marsh-Harrier (*Circus aeruginosus*) 1, Common Wood-Pigeon (*Columba palumbus*) 1, Eurasian Cuckoo (*Cuculus canorus*) 1, Peregrine Falcon (*Falco peregrinus*) 1, Eurasian Coot (*Fulica atra*) 1, Common Moorhen (*Gallinula chloropus*) 1, Black-winged Stilt (*Himantopus himantopus*) 1, Black-crowned Night Heron (*Nycticorax nycticorax*) 1, Eurasian Golden Oriole (*Oriolus oriolus*) 1, *Parus* spp. 1, Greater Flamingo (*Phoenicopterus ruber*) 1, Eurasian Woodcock (*Scolopax rusticola*) 1, European Serin (*Serinus serinus*) 1, Tawny Owl (*Strix aluco*) 1, Blackcap (*Sylvia atricapilla*) 1, Eurasian Hoopoe (*Upupa epops*) 1, Northern Lapwing (*Vanellus vanellus*) 1.

Another 245 birds, belonging to 68 species, were passively collected: 332 specimens (79.4%) came from wildlife rehabilitation centers (WRCs), and 86 (20.6%) were found dead in the field (Table 4). The most commonly passively collected birds were 40 Eurasian Collared-Doves (*Streptopelia decaocto*), 37 Eurasian Blackbirds (*Turdus merula*), 35 Common Kestrels (*Falco tinnunculus*), and 29 Little Owls (*Athene noctua*).

### Detection and isolation of viruses

The viruses were not detected in overwintering mosquitoes or in the mosquitoes sampled early in the season. Assuming a potential USUV minimum infection rate of 0.4, obtained with the 2010 data (213,235 *Cx. pipiens* specimens sampled from the sampling of the first USUV-positive pool and 89 USUV-positive pools), at least one USUV-positive mosquito was expected among the 3,524 overwintering *Cx. pipiens* mosquitoes sampled.

In 2011, all mosquito pools tested negative for WNV, while 74 *Cx. pipiens* pools, six *Ae. albopictus* pools, one *Ae. caspius* pool, and one *An. maculipennis s.l.* pool were USUV positive (Table 3, Figure 1). Fifty-four of these pools were sampled with plan traps and 28 with Modena traps.

**Figure 2:**
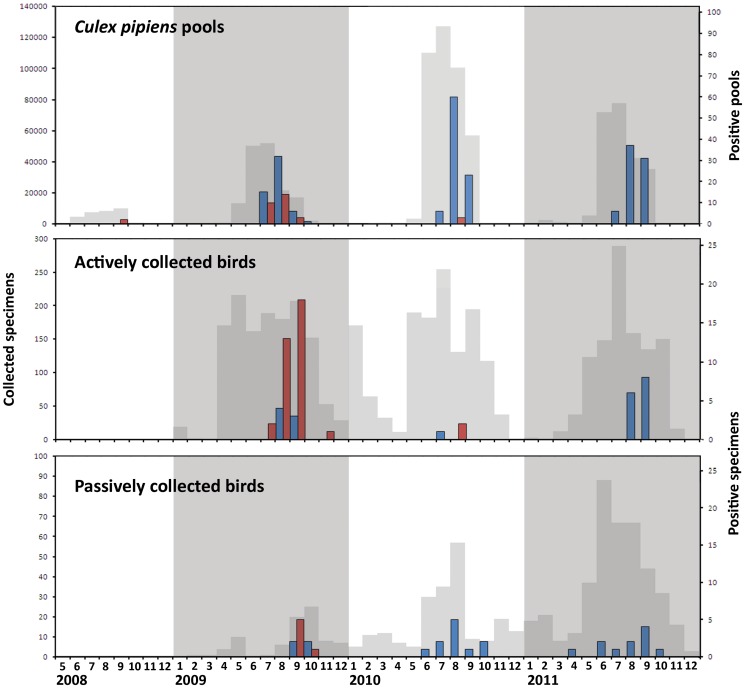
Number of collected (grey) and WNV-positive (red) and USUV-positive (blue) *Culex pipiens* pools, actively collected birds, and passively collected birds.

The first *Cx. pipiens* USUV-positive pool was sampled in the province of Bologna on July 14, and the last was sampled in the province of Modena on September 30 (Figure 2). The first *Ae. albopictus* USUV-positive pools were sampled on July 22 and the last on October 4, both in the province of Modena. The positive *An. maculipennis s.l.* pool and the *Ae. caspius* pool were sampled on September 13 with a Modena trap.

Flavivirus sequences were detected in 22 *Ae. albopictus* pools and in three *Ae. caspius* pools; these sequences had a high identity rate for *Aedes* flavivirus and *Ochlerotatus* flavivirus, respectively, two mosquito-only flaviviruses previously reported in Italy [17].

No birds tested positive for WNV; however, 25 were USUV positive. Of these birds, 14 were collected in active surveillance, and 11 were collected in passive surveillance. Almost all positive birds collected in active surveillance were Magpies (13/14), and one was a Eurasian Jay. Of these birds, six birds were sampled in Bologna province, five were sampled in Ravenna province, and three were sampled in Ferrara province. In passive surveillance, one Magpie was positive for USUV. Of the other birds passively collected, two Starlings and eight other different species were positive (Table 4). None of these birds was found dead on the field. The permanence time in the WRC was available for eight birds. Only a Collared-Dove (*Streptopelia decaocto*) was hospitalized for 10 days, while the other died within two days after admission, including the Greater Spotted Eagle (*Aquila clanga*) that was recovered in poor condition and died shortly after admission. Of these birds, seven came from Ferrara province, two from Reggio Emilia province, and two from Rimini province. The first bird to test positive for USUV was the Greater Spotted Eagle collected in Reggio Emilia province on April 12, and the last was a Starling collected on October 18. Both birds died at a WRC shortly after admission.

USUV was successfully isolated from a Blackbird that died at a WRC in Ferrara province on September 22 and from a *Cx. pipiens* pool of mosquitoes (47 specimens) that were sampled in Rimini province on September 11.

### USUV sequence

The 42 sequences obtained with the flavivirus-genus PCR showed an identity range of 99.1% to 100% out of 214 positions compared. Compared to the 2009 sequence, the 2011 sequence differed by one base pair (bp) and by another base pair compared to the 2010 consensus sequences (out of 264 bp compared). Interestingly, one of these mutations was present in the 2010 and 2011 partial NS5 consensus sequences, but was not present in the 2009 consensus sequence or in any other GB sequence (G>A in genomic position 9060 of EF206350). This mutation could have been acquired while the virus was expanding.

The 55 sequences obtained with USUV-specific PCR showed inter-identity ranging from 99.4% to 100%, out of 361 positions compared. The 2011 PCR consensus sequences were identical to the 2009 and 2010 consensus sequences obtained from the mosquitoes and birds sampled in the Emilia-Romagna region [2], [3]. There were no mutations in the obtained sequences; therefore, no changes were observed in the sequence of the translated proteins.

### Surveillance results from 2009 to 2010

#### Mosquitoes

In March 2009 and 2010, overwintering mosquitoes were sampled manually from winter hibernacula. In 2010, 894 specimens were collected (of which 764, equal to 85.0%, were *Cx. pipiens*) at 50 sites selected within a 5 km radius from stations that had at least one positive mosquito pool in 2009. In 2009, 991 female specimens (of which 512, i.e., 51.7%, were *Cx. pipiens*) (Table 1) were collected in 28 suitable sites in areas with WNV-positive horses, birds, and mosquito pools in 2008 [1], [16]. The different ratio between the two species recorded in this year was probably due to the high density of *An. maculipennis* in the sampled area and to the wide seasonal variations in the density of the two species, which may also affect the number of overwintering specimens. Early seasonal samples were collected at 48 sites in April 2009, and 2,587 mosquitoes were collected. A high proportion of these were *Cx. pipiens* (2,040 specimens, 78.9% of the total) (Table 2).

In 2010, 438,558 mosquitoes collected between May and October were tested [2], 190,516 in 2009 [3] and 47,453 in 2008 [16]. Of these mosquitoes, *Cx. pipiens* was the most abundant species, with 585,297 specimens and an overall rate of 86.5% (90.9% in 2010, 81.4% in 2009, 66.2% in 2008).

#### Birds

In 2010, a total of 1,596 birds were tested: 1,385 were actively collected, and 211 were obtained passively. Of these birds, 157 (74.4%) were from WRCs, and 54 (25.6%) were found dead in the field (Table 5). In 2009, 1,464 birds were tested: 1,378 were actively collected, and 86 were passively collected. Of these birds, 60 (69.8%) were from WRCs, and 26 (30.2%) were found dead in the field (Table 5). These results are partially reported in Calzolari *et al.* [2] and Tamba *et al.* [5].

**Table 5:** Summary of positive samples in the four-year survey.

	2008	2009	2010	2011
Mosquitoes				
*Cx. pipiens* pools	387	1259	2367	1632
WNV-positive (%)	2 (0.5)	27 (2.1)	3 (0.1)	0
USUV-positive (%)	–	54 (4.3)	89 (3.8)	74 (4.5)
*Ae. albopictus* pools	86	108	131	192
USUV positive (%)	–	2 (1.9)	2 (1.5)	6 (3.1)
Birds				
Active surv. tested		1378	1385	1068
WNV-positive (%)		34 (2.5)	2 (1)	0
USUV-positive (%)		7 (0.5)	1 (0.1)	14 (1.3)
Passive surv. tested		86	211	418
WNV-positive (%)		6 (7)	0	0
USUV-positive (%)		4 (4.7)	11 (5.2)	11 (2.6)

Data partially published in [3], [5], [16], [2].

#### Detection of viruses

Neither virus was detected in the overwintering mosquitoes tested in 2009 and 2010.

Cumulatively, WNV was detected in 32 *Cx. pipiens* pools in mosquitoes sampled from 2008 to 2010 (two in 2008, 27 in 2009, three in 2010) (Table 5). USUV was detected in 2009 and 2010 in 153 *Cx. pipiens* pools (54 in 2009 and 89 in 2010) and in four *Ae. albopictus* pools (two in 2009 and two in 2010) (Table 5). WNV was detected in 40 birds in 2009 and in two birds in 2010. USUV was detected in 11 birds in 2009: seven actively collected and four passively collected (of which two Blackbirds found dead in the field). USUV was detected in 12 birds in 2010, one actively collected and 11 passively collected (of which two Blackbirds found dead in the field) (Table 5, Table S1). The permanence time in a WRC was recovered for six of these birds. All died within two days after arrival. Positive bird species are reported in Table S1. The details of and results for the mosquitoes collected from May to October in 2008–2010 and birds collected from May to October in 2010 and from May to November in 2009 have been reported in previous publications [2], [3], [5], [16].

#### Estimation of reservoir importance of bird populations

An estimation of the relative reservoir importance of birds tested in active surveillance was performed using 2009 data, a year with a relevant WNV circulation. The infection rate of Magpies, Crows, Jays and Starlings was found by utilizing data from surveyed areas, and then the number of potential infected specimens were estimated according to the bird abundance (Table 6). In the 2009 scenario, among the four actively collected species, Starlings seemed to be the more important WNV-reservoir hosts, followed by Magpies. Due to the low number of USUV-positive birds actively collected, this estimation was not performed for USUV.

**Table 6:** Estimation of actively collected bird populations and WNV infected specimens according the 2009 data from the surveyed area.

	Estimated population (% collected)	*n*/WNV+	Infection rate	Estimation of infected birds
Common Starling	479,000 (0.02)	88/5	5.68	27,200
Eurasian Magpie	52,000 (1.36)	707/23	3.25	1,700
Hooded Crow	48,000 (0.90)	429/4	1.17	600
Eurasian Jay	19,000 (0.49)	94/1	1.06	200

Data partially published in [5].

## Discussion

**Figure 3:**
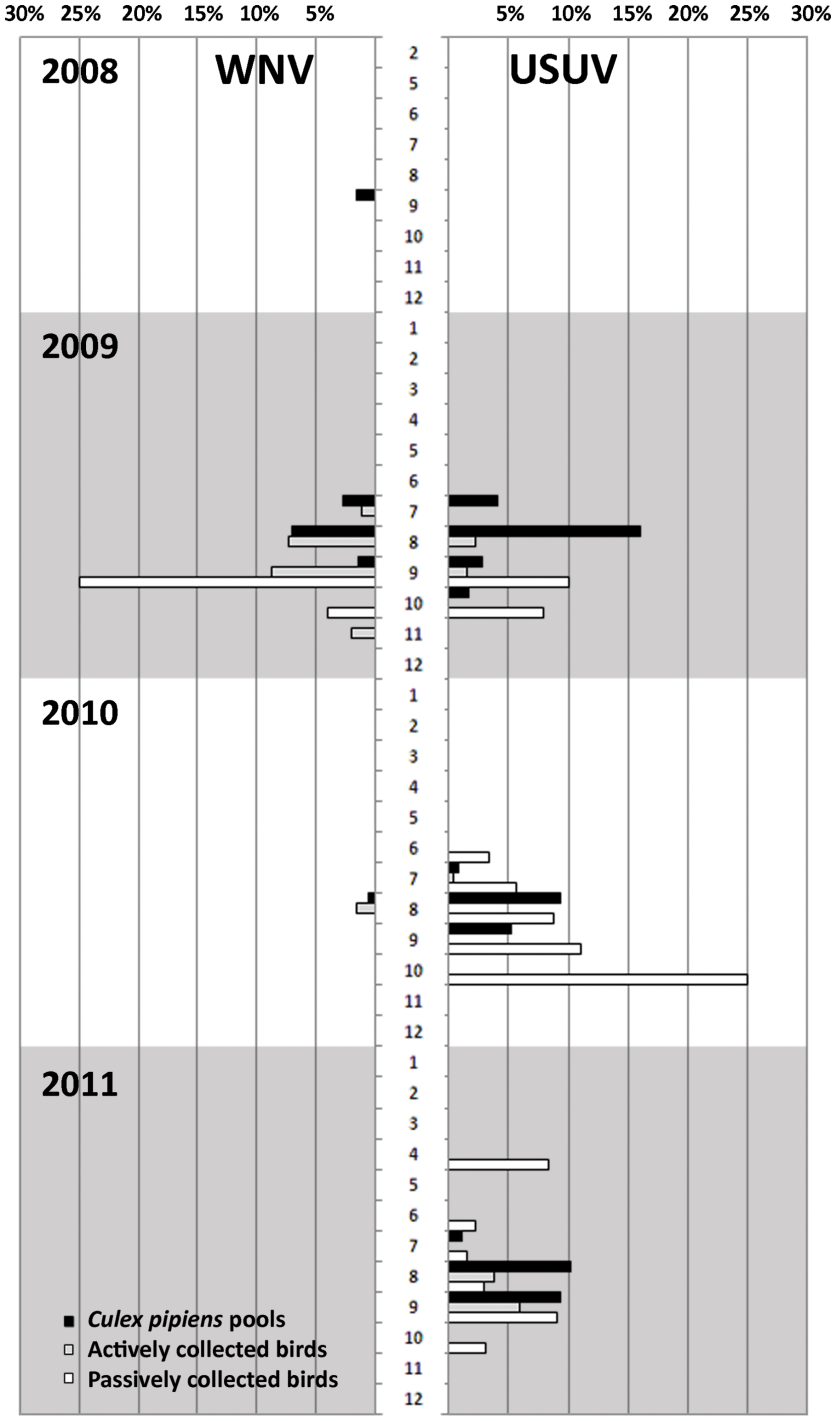
Percentage of positive *Culex pipiens* pools and actively and passively collected birds for the survey month in different years.

WNV and USUV showed different patterns of activity during the surveillance period 2008–2011 in the Emilia-Romagna region. WNV was detected in 2008, 2009, and 2010, but in 2011 no positive birds or mosquitoes were reported. However, USUV was continuously active throughout 2009, 2010, and 2011 (Figure 2, Figure 3). Despite the limited length of the 2011 USUV sequences, obtained with the two diagnostic PCRs, the high identity with previous year sequences suggests that the virus overwintered in the region [2]. Previous data also strongly sustain the hypothesis of overwintering for WNV between 2008 and 2010 [2], [3], [4]. The geographic distribution of the viruses in the territory over several years of surveillance also supports the hypothesis of overwintering of both viruses, rather than different reintroductions.

A possible mechanism of arbovirus overwintering in temperate climates could be through infected diapausing mosquitoes [14], [18]. USUV-positive mosquitoes in diapause have been reported in Europe [19]. Furthermore, WNV-positive overwintering mosquitoes were detected several times in the United States [20], [21], [22], [23], but always at low rates, and often after seasons with a high infection rate in mosquitoes [23]. Moreover, a reduced overwintering capacity was recorded in engorged mosquitoes [24], and various field observations reported a low parous rate in mosquitoes in diapause, which decreased during the winter [25], [26], often reaching zero at the new season start [27]. In addition to this scarce probability of virus overwintering in directly infected diapausing mosquitoes, vertical transmission could be another mechanism supporting WNV overwintering. Vertical transmission of the virus has been shown by WNV detected in mosquito larvae sampled in the field [28] and in adults reared from field-collected larvae [17], [29]. However, a low rate of vertical transmission was often found in WNV experimental infection of mosquitoes [30], [31], [32], [33], [34], [35], suggesting that this phenomenon is rare in nature.

Furthermore, if virus overwintering did occur in mosquitoes, it would have to occur in enough infected mosquitoes to restart viral circulation in the following season. This number must be sufficient to ensure the passage of the virus to a competent host, since a female mosquito feeds a limited number of times, to mature the eggs. Limited presence of blood from different hosts was found in engorged *Cx pipiens* mosquitoes, which seems to demonstrate that multiple feeding is a rare event for this species [36], [37], [38], and therefore limits the capacity of this species to transmit the virus. During the three years of surveillance, two overwintering mosquito species were sampled more abundantly, *Cx. pipiens* (80.1%) and *An. maculipennis s.l.* (19.8%), but none tested positive, despite the large sampling effort expended in areas where positive mosquito pools had been sampled the previous summer. Furthermore, the first positive mosquito pool was recorded in July, even though mosquito sampling was performed early in the season (Table 2) and the majority of sampling started in June. The data suggest that diapausing mosquitoes were not the main source of virus overwintering in the Emilia-Romagna region; another mechanism probably exists.

Vertebrate hosts could also provide a way for viruses to overwinter. Long-term viremia was described in the House Sparrow [39]. WNV was detected in the urine of human convalescent patients [40] and in the kidneys of laboratory hamsters [41] months after infection, showing the virus persists for long periods in some organs. Ingestion of infected animals by susceptible hosts, by hunting or carrion-feeding behavior, may be an alternative virus transmission method [13], [14], [39], [42]. The mortality of birds at the end of winter could facilitate this mechanism of infection in opportunistic scavengers, such as European Magpies and Hooded Crows, and, therefore, WNV overwintering in birds.

The relevance of this mechanism in WN and USU viruses overwintering in Emilia-Romagna seems to be confirmed by finding birds that tested positive for the viruses early in the season, when the mosquito density was low, as the Spotted Eagle died on April 12, 2011, and tested USUV-positive, and the WNV-positive magpie was sampled in May 2009 [5].

The surveillance system reported an increase in WNV circulation from 2008 to 2009, with a successive decrease in 2010, and failed to detect the virus in 2011 (Table 5), despite comparable sampling effort. Herd immunity within the reservoir bird populations could explain the drastic drop in WNV circulation, as already reported [43]. In effect, durable and protective WNV immunity has been described for various bird species [44].

This reported decline in WNV circulation is consistent with the dynamic observed in many European countries, with a progressive decrease in WNV activity in the years after the first onset of an outbreak [45], [46]. These reports and our results are comparable with a peri-Mediterranean circulation of WNV lineage 1 related strains, which disappeared from an area, or circulated into *nidus* at an undetectable sylvatic cycle, after about two to three years of epidemic circulation, perhaps due to the trigger of herd immunity, and are probably transported to a new territory by short-ranging migrant birds, not necessarily from Sub-Saharan Africa.

Eurasian Magpies, Hooded Crows, and Eurasian Jays tested during the survey represent a relevant part of the estimated population of these birds in the surveyed area (Table 6). In addition, an relevant number of Common Starlings was tested, of which five tested WNV-positive in 2009 [5] (Table S1). Crows are more abundant in Emilia-Romagna western part and in specific environments, like poplar plantations, while Magpies are more abundant in eastern part of the region, especially in particular plain environments [47]. Although the three corvids are sedentary in Emilia-Romagna, Common Starlings have a different tendency to migrate, and large part of the regional population is sedentary [47], [48]. These birds belong to the Passeriformes order, and a primary reservoir role was recognized for passerines from experimental studies [13] and European field data [49]. Moreover, these birds are abundant in Emilia-Romagna, so they are good candidates for WNV-reservoir hosts. According to the abundance estimation and 2009 data, a more important reservoir role between these species could be assigned to Starlings and Magpies (Table 6). These two species are more abundant in the plains and prefer arable land and urban area compared to Crows and Jays, which prefer woodland areas, and are more common at higher altitude [47], [50]. According to the *habitat* preference of Starling and Magpie, the number of mosquito WNV-positive pools was significantly higher in rural areas in 2009 [3], confirming the greater circulation of this virus in a rural environment.

Moreover, the observed two- to three-year decline in WNV circulation is in accordance with the 90% turnover in the population of possible bird reservoirs, such as the European Starling, which was estimated with ringing activity [48]. This is consistent with a primary role in the WNV herd immunity mechanism carried out by hatch year birds, which has been reported in the United States [51]. The high rate of immunization in hatch year birds could be due to greater exposure to mosquito bites, higher sensitivity to the virus, and the possible direct passage of the virus to the nestling, as mechanisms of passive immunity from mother to offspring exist but seem to have a short duration [52].

In contrast to WNV, the circulation of USUV did not show evidence of a decrease. After the virus in 2009 was first detected, the surveillance system constantly detected this virus in mosquitoes and birds until 2011, with similar prevalence in different years (Table 5). Possible cross immunization between USUV and WNV could influence this dynamic. However, birds tested positive for both viruses, which was reported in the Emilia-Romagna region [2], [5] seems to rule out this hypothesis. The absence of a decrease in the circulation of USUV (Table 5) could be due to a different mechanism of immunity in birds for the two viruses; however, a USUV herd immunity phenomenon was described in Austria [53]. If this is the case, the persistence of this virus could be explained by not yet identified reservoirs, in addition to wild birds, unable to develop sufficient herd immunity due to an ineffective immune response or because of a fast population turnover, as in the case of rodents [14].

A possible difference in the epidemiology of the two viruses seems also supported by the detection of USUV in *Ae. albopictus* pools (obtained in 2009, 2010, and 2011) and *An. maculipennis* and *Ae. caspius* pools in 2011. These findings do not necessarily confirm the USUV vectorial competence of these species, but the feeding habits of these mosquitoes may indicate the possible involvement of some mammals in the cycle of this virus. *Ae. albopictus* is a marked opportunistic feeder mosquito, whereas species of the *An. maculipennis* complex and *Ae. caspius* mosquito are mostly mammophilic [54], [55].

The level of USUV circulation detected by the surveillance system in 2011 (Table 5) could not be explained by the mosquito abundance recorded, lower in 2011 than in 2010, indicating a weak correlation between mosquito density between years and USUV circulation, as previously observed in Emilia-Romagna [2]. This confirms that a high density of vectors does not automatically mean higher viral circulation [2] as other factors may influence the arboviral load in the environment, such as the level of immunity in the reservoirs and several environmental parameters. The dynamics of the two viruses are similar in all years, with the circulation peaking 1-2 weeks earlier in mosquitoes than in birds (Figure 3), which seems to confirm the primary role of mosquitoes in amplifying the viruses already present in reservoirs.

Reported data highlighted differences in the epidemiology of the two viruses but leaves open several questions, and points out the arbovirus ability to exploit complex interactions between species to persist in the ecosystem, indicating that only a complete understanding of the ecology can explicate its maintenance mechanisms. This is also true in the highly anthropized environment, typical of the surveyed area. In fact, the success of an arbovirus could be linked to the simplification of the ecosystem, which causes an exponential increase in several species, with a consequent increase in their reservoir potential. This is in accord with reports that indicate a rise in the burden of vector-borne diseases as biodiversity decreases [56], [57], implying a possible buffer role of biodiversity on the impact of these diseases.

## Materials and Methods

### Ethics statement

Mosquitoes were sampled from public land according to the guidelines of the notes of the Regional Health Authority (RHA) (Notes PG/2009/128190 June 5, 2009, PG/2010/194887 July 30, 2010, PG/2011/167352) that provided implicit authorization to perform the sampling on public land. Mosquitoes sampled from private land were collected after verbal informed consent was received from the landowners.

The actively sampled animals were shot or captured by authorized personnel under the specific wildlife population control program approved by Istituto Superiore per la Protezione e la Ricerca Ambientale (ISPRA). Tests for WNV/USUV detection were performed according to the RHA notes (PG/2011/91006 of April 11, 2011, PG/2010/93237 of March 31, 2010, PG/2009/50028 of February 27, 2009). These notes include ISPRA authorization to test these birds. Birds found dead were collected through Provinces' Guards or Veterinary Services Officers and then conveyed to our Institute according to the RHA notes (PG/2011/91006 of April 11, 2011, PG/2010/93237 of March 31, 2010, PG/2009/50028 of February 27, 2009). Dead birds from WRCs were collected by Veterinary Services Officers and transported to IZSLER for diagnostic purposes listed in the RHA notes, including WNV and USUV monitoring (PG/2011/91006 of April 11, 2011, PG/2010/93237 of March 31, 2010, PG/2009/50028 of February 27, 2009).

### Survey area

The survey area is part of the Po valley administratively in the Emilia-Romagna region, approximately 12,000 km^2^. This area is the largest Italian floodplain, characterized by intensive agriculture and animal husbandry. The eastern part of the area is on the Adriatic Sea and is marked by large natural wetlands (Valli di Comacchio and Po River Delta). The climate is typically sub-continental, gradually becoming sub-Mediterranean toward the coastal part of the region. All of the monitored territories are densely populated, with cities, villages, and industrial areas. Industrial activity is highly developed, and the Pianura Padana is strongly influenced by human activity, characterized by intensive agriculture, with a few hedges, scattered natural protected areas, and a dense irrigation network. Non-cultivated zones are rare (e.g., riverbeds, disused quarries, newly established wetlands). The most common crops are cereals and maize; vineyards or orchards are locally abundant, and poplar cultivation is widespread in flood zones near rivers. Pinewood and Mediterranean thicket vegetation is typical of the coastal part of the region.

### Mosquito collection

From February to March, hibernating mosquitoes were manually sampled in 43 suitable places, sited in a radius of 5 kilometers (km) from stations with at least a positive mosquito pool in the previous season. The locations were typical places where mosquitoes hibernate, such as damp rooms of old brick buildings, especially basements. The mosquitoes were collected when they were leaving their hibernacula with a large battery-powered backpack aspirator or a small hand-held mechanical aspirator.

Host-seeking mosquitoes were collected with modified CDC traps baited with CO_2_ (CO_2_ traps) [58]. All traps were georeferenced and operated from approximately 4:00 p.m. to 10:00 a.m.

Sampling sites were managed with various methods: (1) Ninety regularly sampled fixed stations, placed in a 10 km grid in the surveyed area, were activated every 14 days from the end of May to the end of September (plan traps). (2) Forty-five stations were monitored every 14 days, from August 2 to October 19, in an area that had intense WNV/USUV circulation in 2009 and 2010 (Modena traps). (3) Mosquitoes collected for other purposes in eight single-night sampling stations, in six municipalities, were also tested (extra-plan traps).

Mosquitoes were identified to the species level using morphological characteristics according to three classification keys [54], [55], [59], [60]. The *Ochlerotatus* taxon was considered an *Aedes* sub-genus [61]. Mosquitoes were pooled according to date, location, and species, with a maximum of 200 individuals per pool [62]. For every species, a maximum of 1,000 specimens were tested per sampling. The pooled mosquitoes were ground and centrifuged, and an aliquot of the sample was collected according to the method reported in Calzolari *et al.* [2].

### Bird collection

Birds were sampled in the plain and hill areas of the region, and birds born in the year were preferentially examined. Programmed sampling was performed from April to October by dividing the surveyed area into 1,600 km^2^ quadrants and collecting 5–10 specimens in each quadrant every month. Passive surveillance of wild birds was carried out on animals found dead in the field or deceased at WRCs.

Birds were autopsied, and organ samples (brain, spleen, heart, and kidney) were pooled, ground, and submitted to biomolecular analysis. Samples from every bird were processed individually.

#### Estimation of bird populations

The estimation of bird populations is difficult, due to the scarcity of data, the fluctuation in the different years and the heterogeneous abundance trough the territory. To obtain a range of breeding couple (*c*) abundance in surveyed area, data provided by MITO2000 (www.mito2000.it) [63] were applied to the estimates of Italian breeding population [64] (Table S2). Moreover couple abundance were assessed through faunal reports [50], [65] and experts’ experience in 1–40 *c*/km^2^ for Magpie, 1–5 *c*/km^2^ for Crow, 0.5–10 *c*/km^2^ for Jay, 5–40 *c*/km^2^ for Starling. According to both estimations, density of 1.3 c/km^2^ was assessed for Magpie, of 1 *c*/km^2^ for Crow, of 0.8 *c*/km^2^ for Jay and of 20 *c*/km^2^ for Starling. Non-breeding individuals were estimated in 40% of total population for Magpie and 50% for Crow and were considered not relevant in Starling and Jay populations.

### Virus survey

The virus survey was conducted according to the methods used in previous season [2]. RNAs present in samples were extracted using TRIzol®LS Reagent (Invitrogen, Carlsbad, CA); cDNA synthesis was achieved using random hexamer (Roche Diagnostics, Mannheim, DE) and SuperScript® II Reverse transcriptase (Invitrogen, Carlsbad, CA) according to the manufacturers’ instructions.

Mosquito and bird samples were analyzed using three different polymerase chain reactions (PCRs): (1) traditional PCR, targeted at the *NS5* gene fragment, to detect flavivirus-genus according to Scaramozzino *et al.* [66]; (2) traditional PCR to detect USUV [67]; and (3) real-time PCR to detect WNV, according to Tang *et al.*’s method [68]. Fragments obtained with PCRs were sequenced with an automated fluorescence-based technique following the manufacturer’s instructions (ABI-PRISM 3130 Genetic Analyzer, Applied Biosystems, Foster City, CA). To obtain WNV sequences, a traditional PCR protocol was performed on WNV-positive samples with the primers described in Lanciotti *et al.* [69], targeted at *C* and *prM* genes.

The sequences were used to perform a basic local alignment search tool (BLAST) in the GenBank library to confirm the specificity of the positive reaction and to estimate the degree of identity of the detected strains. The sequences were compared with available GenBank sequences using the ClustalW alignment algorithm in the freeware program MEGA 5 [70]. All sequences are available from the authors upon request.

Virus isolation was attempted starting from the remaining part of the PCR-positive mosquito homogenates using the Vero cell line (American Type Culture Collection, Rockville, MD), incubated at 37°C, and the C6/36 cell line [71], incubated at 28°C.

### Statistical analysis and point pattern analysis

All the collected data were managed by a dedicated Microsoft Access database. Due to the non-normal distribution of the dataset, the abundance of mosquitoes collected during 2009–2010 was tested using the non-parametric Kruskal-Wallis test, with p<0.05. Statistical analyses were performed using Intercooled Stata 7.0 software (Stata Corporation, College Station, TX).

## Supporting Information

Table S1 **Positive birds for WNV and USUV collected in active and passive surveillance (A/P) in the different years of survey.**
(DOC)

Table S2 **Estimation of breeding couples (** ***c*** **) of actively collected birds in surveyed area of Emilia-Romagna according to 2010-2011 MITO2000 data** [**62**] **and** [**63**] **.**
(DOC)
