# Supplementary material for: Usutu Virus Persistence and West Nile Virus Inactivity in the Emilia-Romagna Region (Italy) in 2011
Source: PLoS One. 2013 May 7;8(5):e63978. doi: 10.1371/journal.pone.0063978 (PMC3646878; doi:10.1371/journal.pone.0063978)
Supplement: Table S1 — Positive birds for WNV and USUV collected in active and passive surveillance (A/P) in the different years of survey. (DOC) [file pone.0063978.s001.doc]

Table S1. Positive birds for WNV and USUV collected in active and passive surveillance (A/P) in the different years of survey.

|  |  | 2009 | | 2010 | | 2011 | | Total | |
| --- | --- | --- | --- | --- | --- | --- | --- | --- | --- |
| Name | Species | WNV/+ specimens (A/P) | USUV/+ specimens (A/P) | WNV/+ specimens (A/P) | USUV/+ specimens (A/P) | WNV/+ specimens (A/P) | USUV/+ specimens (A/P) | WNV/+ specimens (A/P) | USUV/+ specimens (A/P) |
| Eurasian Magpie | *Pica pica* | 23 (23/0) | 4 (4/0) | 1 (1/0) | 1 (1/0) |  | 14 (13/1) | 24 (24/0) | 19 (18/1) |
| Common Starling | *Sturnus vulgaris* | 5 (5/0) | 1 (1/0) |  |  |  | 2 (0/2) | 5 (5/0) | 3 (1/2) |
| Hooded Crow | *Corvus cornix* | 5 (5/0) |  |  |  |  |  | 5 (5/0) |  |
| Eurasian Jay | *Garrulus glandarius* | 2 (1/1) | 2 (2/0) | 1 (1/0) | 1 (0/1) |  | 1 (1/0) | 3 (2/1) | 4 (4/0) |
| Seagulls | *Larus spp.* | 3 (0/3) | 1 (0/1) |  |  |  | 1 (0/1) | 3 (0/3) | 2 (0/2) |
| Long-eared Owl | *Asio otus* | 1 (0/1) |  |  | 2 (0/2) |  |  | 1 (0/1) | 2 (0/2) |
| Little Owl | *Athene noctua* | 1 (0/1) |  |  |  |  |  | 1 (0/1) |  |
| Eurasian Blackbird | *Turdus merula* |  | 3 (0/3*) |  | 5 (0/5*) |  | 1 (0/1) |  | 9 (0/9) |
| Eurasian Collared-Dove | *Streptopelia decaocto* |  |  |  | 1 (0/1) |  | 1 (0/1) |  | 2 (0/2) |
| House Sparrow | *Passer domesticus* |  |  |  |  |  | 1 (0/1) |  | 1 (0/1) |
| Eurasian Green Woodpecker | *Picus viridis* |  |  |  |  |  | 1 (0/1) |  | 1 (0/1) |
| European Bee-eater | *Merops apiaster* |  |  |  |  |  | 1 (0/1) |  | 1 (0/1) |
| Grey Heron | *Ardea cinerea* |  |  |  |  |  | 1 (0/1) |  | 1 (0/1) |
| Greater Spotted Eagle | *Aquila clanga* |  |  |  |  |  | 1 (0/1) |  | 1 (0/1) |
| Partridge | *Alectoris rufa* |  |  |  | 1 (1/0) |  |  |  | 1 (0/1) |
| Eurasian Nightjar | *Caprimulgus europaeus* |  |  |  | 1 (0/1) |  |  |  | 1 (0/1) |
| Total |  | 40 (34/6) | 11 (7/4) | 2 (2/0) | 12 (1/11) |  | 25 (14/11) | 25 (36/6) | 48 (23/25) |

* Two specimens collected death in the field.
