# Supplementary material for: Usutu Virus Persistence and West Nile Virus Inactivity in the Emilia-Romagna Region (Italy) in 2011
Source: PLoS One. 2013 May 7;8(5):e63978. doi: 10.1371/journal.pone.0063978 (PMC3646878; doi:10.1371/journal.pone.0063978)
Supplement: Table S2 — Estimation of breeding couples ( c ) of actively collected birds in surveyed area of Emilia-Romagna according to 2010-2011 MITO2000 data [62] and [63] . (DOC) [file pone.0063978.s002.doc]

**Table S2. Table S2. Estimation of breeding couples (*c*) of actively collected birds in surveyed area of Emilia-Romagna according to 2010-2011 MITO2000 data [62] and [63].**

|  | Italy  (301,336 km^2^) | | Emilia-Romagna  (22,117 km^2^) | | Surveyed area  (11,975 km^2^) | | *c*/km^2^ | |
| --- | --- | --- | --- | --- | --- | --- | --- | --- |
|  | Abundance^a^ | *c*^b^ | Abundance^a^ | *c*^c^ | Abundance^a^ | *c*^c^ | Range | GM |
| Common Starling | 25.5 | 1,000,000-3,000,000 | 82.22 | 235,400-706,100 | 99.53 | 154,300-462,800 | 12.9-38.6 | 22.3 |
| Black-billed Magpie | 5.8 | 200,000-500,000 | 6.15 | 15,400-38,500 | 7.14 | 9,700-24,200 | 0.8-2.0 | 1.3 |
| Hooded Crow | 12.7 | 110,000-520,000 | 9.17 | 5,800-27,400 | 9.70 | 3,300-15,700 | 0.3-1.3 | 0.6 |
| Eurasian Jay | 2.7 | 200,000-400,000 | 2.66 | 14,500-29,100 | 2.20 | 6,500-13,000 | 0.5-1.1 | 0.8 |

^a^ 2010-2011 MITO2000 data expressed in individuals on 10 points of observation, 14108 total points in Italy, 827 total points in Emilia-Romagna of which 676 in surveyed area.

^b^ According to [63]

c
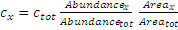


GM: Geometric mean of extreme values
